# Stimulating training and access to HIV research experiences (STAR program): a protocol for a crowdsourced, project-based, implementation science training program using mixed-methods design

**DOI:** 10.3389/fpubh.2025.1586168

**Published:** 2025-09-11

**Authors:** Juliet Iwelunmor, Idia Thurston, Collins Airhihenbuwa, Weiming Tang, Ucheoma Catherine Nwaozuru, Onyekachukwu Anikamadu, David Oladele, Bryce Takenaka, Khadijah Ameen, Olufunto A. Olusanya, Temitope Ojo, Joseph D. Tucker

**Affiliations:** ^1^Division of Infectious Diseases, John T. Milliken Department of Medicine, School of Medicine, Washington University in St. Louis, St. Louis, MO, United States; ^2^Department of Health Sciences and Applied Psychology, Northeastern University, Boston, MA, United States; ^3^Department of Policy and Behavioral Sciences, School of Public Health, Georgia State University, Atlanta, GA, United States; ^4^Department of Medicine, Division of Infectious Diseases, University of North Carolina, Chapel Hill, Chapel Hill, NC, United States; ^5^Department of Implementation Science, Wake Forest University School of Medicine, Winston Salem, NC, United States

**Keywords:** HIV, youth, young adults, HIV research training, crowdsourcing, project-based learning, implementation science

## Abstract

**Background:**

HIV is more common among underrepresented minority adolescents and young adults (AYA, 13–24 years old) in the United States. Low uptake of HIV prevention services suggests a missed opportunity for implementing evidence-based interventions such as pre-exposure prophylaxis (PrEP) and sexually transmitted infection testing among this important population. Most research institutions in the United States also have limited opportunities for AYA training, mentorship, and capacity-building activities.

**Methods:**

The “Stimulating Training and Access to HIV Research Experiences” (STAR) program brings together a highly qualified group of research mentors to achieve three specific aims: (1) identify and recruit underrepresented minority AYA interested in HIV research for STAR using crowdsourcing; (2) develop implementation science research and project-based design capacity for underrepresented trainees at participating US institutions; and (3) initiate and sustain enduring AYA research capacity through a digital participatory learning community. A three-stage approach is taken to increase the number of racial and ethnic minority trainees that: (1) learn about HIV prevention services; (2) lead the design of HIV prevention services; and (3) launch and evaluate HIV prevention services serving UREM AYAs at participating community sites. Furthermore, we create a Participatory Learning Community (PLC), with virtual opportunities for interaction, mentoring, and sharing of project-based designs so that rapid exchanges can occur and be documented among trainees, faculty, and invited scholars in the field.

**Discussion:**

There is a substantial unmet need for adolescent and young adult (AYA) HIV implementation research training in the United States among underrepresented minority AYAs. STAR seeks to identify highly qualified trainees through open calls, build capacity for youth-led research using designathons and innovation bootcamps, and sustain these benefits through participatory learning communities. These approaches break new ground in HIV training using participatory methods that empower AYAs to become junior leaders while building institutional capacity for AYA HIV research.

## Introduction

Nearly 1 in 5 people who were newly diagnosed with HIV are adolescents and young adults (AYA) between the ages of 13 and 24 (1, 2). People in the AYA age group are least likely to be aware of their HIV serostatus due to low HIV testing rates (1). Further, most do not access essential prevention services such as pre-exposure prophylaxis (PrEP) or sexually transmitted infection (STI) screening (3). An estimated 700,000 AYA in the US could benefit from PrEP, but only 27,330 PrEP prescriptions have been written for this population since 2012 (1). Similarly, although AYA represent approximately 25% of the sexually active population in the US, most are not routinely screened for STIs. A national survey among AYA found that only 16.6% of women/girls and 6.6% of men/boys received an STI test in the past 12 months (4). *The poor uptake and linkage to essential youth-friendly HIV prevention services in the US present a critical gap in HIV prevention efforts in the US.* We define essential HIV prevention services as HIV testing (including self-tests and facility-based testing), gonorrhea/chlamydia/syphilis testing, STI treatment among those with infection, PrEP initiation, and 100% condom use. We define youth-friendly as serving the unique needs, preferences, developmental capacity, and life stages of youth (5). Youth-friendly HIV prevention services that ensure screening and testing of HIV and other STIs remain a national priority (*Goal 1 of the US HIV National Strategic Plan 2021–2025*) (6, 7).

Conventional HIV interventions are often top-down, expert-driven (i.e., one-size-fits-all) processes, with few opportunities for input from Underrepresented Racial and Ethnic Minority (UREM) AYA (8, 9). This results in both technical and substantive problems. The inclusion of UREM AYA in the process and governance of HIV research can create a more effective, engaging, and equitable outcome in research (9, 10). One promising participatory engagement method with AYA is a crowdsourcing open call (11, 12). Crowdsourcing is a participatory approach to solicit ideas from large groups of diverse individuals, which provides an opportunity for youth themselves to foster innovative and youth-informed ideas (11). Open calls provide a structured mechanism to aggregate insights and wisdom from AYA directly in response to a specific problem, leading to innovative solutions that are then shared with the public (5). Open calls can help to access AYAs that would otherwise be difficult to engage (13–15). The US Office of Science and Technology Policy identified open calls as a centerpiece of the US Strategy for American Innovation, and the America COMPETES Reauthorization Act gives all government agencies the broad authority to conduct open challenges to promote innovation (16, 17). *HIV training programs that use bottom-up strategies, such as crowdsourcing, offer a unique opportunity to reduce HIV-related disparities and health inequities (Goal 3 of the HIV National Strategic Plan 2021–2025)* (7).

Racial ethnic minorities make up over 30% of the US population, but less than 9% of people are in health and biomedical professions (18, 19). This low percentage is problematic, as a diverse workforce is integral to ending the HIV epidemic by 2030. Research training and mentoring for students from underrepresented backgrounds are particularly valuable because the “ripple effects” can extend beyond individual students’ careers to their peers, family, community, and institutional environments. This benefits individuals, research teams, and institutions (20). Without significant investment in UREM AYA, the United States is unlikely to achieve its goals for diversity in the biomedical research workforce.

Increasing the size and diversity of the workforce in HIV intervention research is critical for achieving the US government, NIH, and NIAID strategic priorities (21). Similar training efforts in adjacent fields, including programs focused on healthcare worker engagement and high-risk populations, have emphasized the importance of tailored mentorship, sustained engagement, and flexible delivery models (22). These insights helped inform elements of the STAR program design, particularly in addressing engagement and capacity-building needs among underrepresented early-career researchers. However, few training models are designed to combine participatory learning, sustained mentorship, and real-world application for underrepresented early career researchers, particularly in the context of HIV.

In response to this need, the Stimulating Training and Access to Research Experiences (STAR) program (STAR) – (R25 AI170379) was launched to support the next generation of HIV researchers from underrepresented backgrounds. Now in its third year, the program is funded by the National Institutes of Allergy and Infectious Diseases through a strategic request for proposals focused on advancing the careers of a diverse research workforce, particularly among undergraduate and graduate student learners. STAR is a collaboration between Washington University in St. Louis, the University of North Carolina, Chapel Hill, Northwestern University, Georgia State University, and Wake Forest University. STAR’s mission is to affect change in the HIV epidemic through participatory learning, discovery, and communication. We strive to nurture voices hidden and unknown, silenced or resilient, creative and yearning, open and curious, bold and fearless, all united with the fierce urgency to end HIV as we know it.

## Methods and design

### Conceptual framework

**Figure 1:**
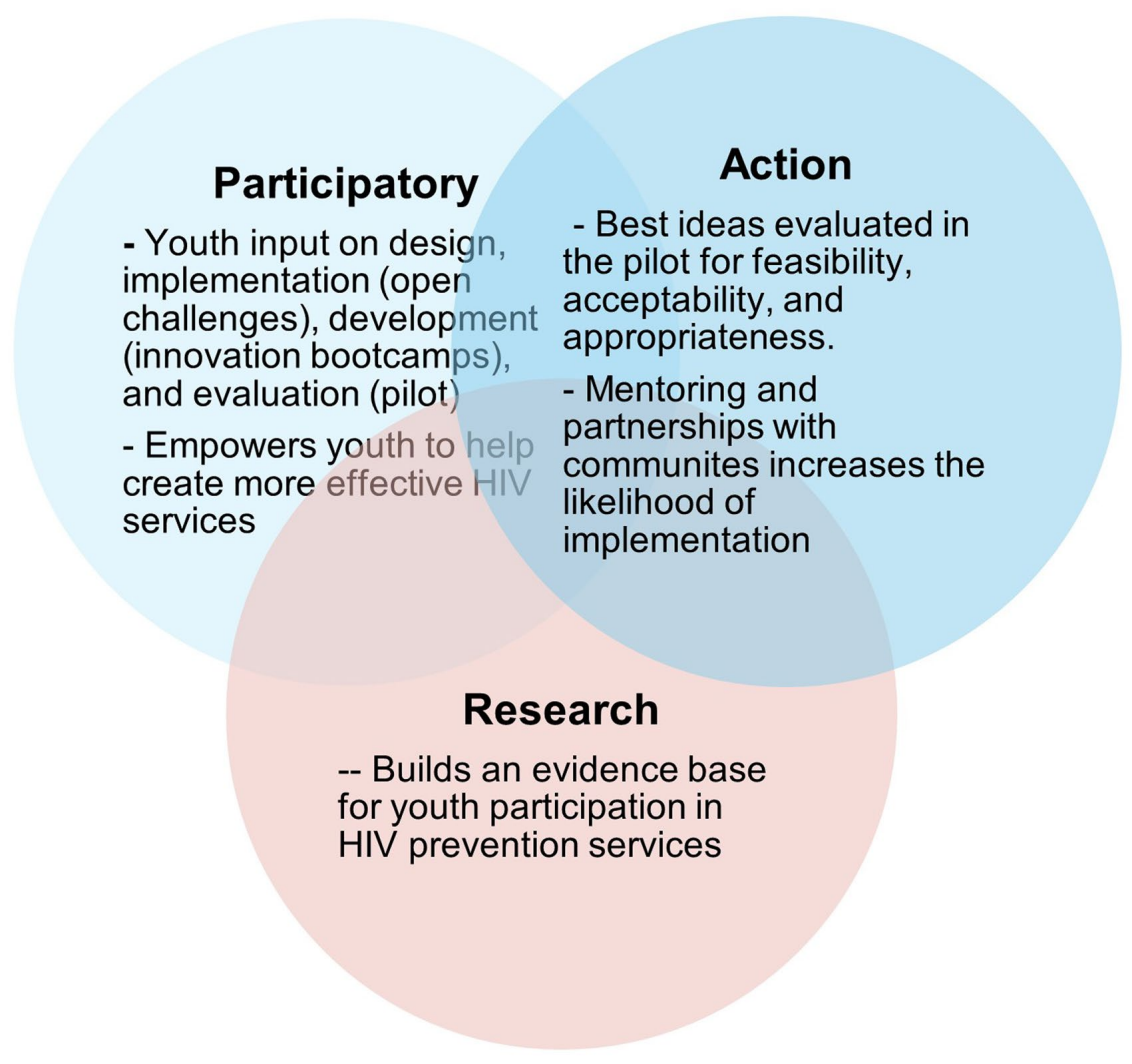
Conceptual framework for the STAR program.

To enhance clarity and reproducibility, we structured the following description of the STAR program to align with key elements recommended in Template for intervention description and replication (TIDieR) and Standards for Reporting Implementation Studies (StaRI), established guidelines for educational and implementation interventions. Our approach is summarized in Figure 1. We utilize youth participatory action research (YPAR) as our conceptual framework in all STAR activities. YPAR provides youth with opportunities to learn about social problems (like HIV) that affect their lives and then propose actions to address these problems (23–26). It considers youth as potential experts and co-creators of knowledge (27). Studies using youth participatory action have improved many HIV-related outcomes (27, 28). YPAR research focuses on self-efficacy and empowerment (29), gender-based violence (30), teen pregnancy (30), community violence (31), and educational outcomes (32). This conceptual framework (Figure 1) directly informs all phases of STAR activities.

### Core content

The STAR training program comprises three main components: (1) Crowdsourcing activities, including open calls (for team recruitment), designathons (to facilitate initial mock-ups of project-based HIV prevention ideas), and a six-week Innovation Bootcamp (six-week program to refine implementation designs further); (2) A six-week Implementation Science Research Learning module and Field-related Activities; and (3) A one-year Participatory Learning Community to facilitate and sustain a STAR network of scholars. Additionally, trainees are paired with faculty mentors and are required to participate in mentoring sessions and engage in a participatory learning community, where they share their project designs with colleagues and mentors to receive feedback.

### The rationale for project-based design and implementation science research training

Despite the availability of evidence-based interventions, few are implemented and sustained at scale for adolescents and young people in the US. Additionally, UREM AYA are not engaged or involved as partners and leaders in the implementation and dissemination of these interventions (33). As a result, tremendous gaps remain along the HIV prevention continuum for UREM AYA (33). This is a missed opportunity to reduce onward transmission to and acquisition of HIV in at-risk youth and to evaluate scalable, evidence-based interventions serving AYA populations (33). Implementation Science studies methods promote the adoption and integration of evidence-based practices, interventions, and policies into routine health care and public health settings. It involves the use of theories, models, and frameworks to optimize study design, data collection, analysis, and dissemination. The promise of implementation science for addressing the HIV burden among AYA, including strengthening a diverse HIV workforce made up of racial and ethnic AYA minorities to end the HIV epidemic, has not been fully realized in the US. Likewise, with the recognition that a one-size-fits-all approach may not fully address the needs of AYA, STAR takes an authentic, project-based learning approach that emphasizes real-world designs by transforming ideas and data into usable, understandable, and appealing health interventions for specific audiences. We deploy a 4-component training bundle guided by the Expert Recommendations for Implementing Change that includes:

1. Train and educate AYA researchers: An open call is conducted to identify and recruit AYA UREM scholars to the STAR program. Students are required to submit entries focused on promoting evidence-based HIV prevention interventions targeting AYA among community-based organizations in the US.
2. Adapt and tailor designs to local context: Once selected, students engage in designathons and innovation bootcamps to tailor their strategies to local contexts.
3. Engage and empathize with end-users: Students are encouraged to empathize more effectively with their audiences by creating fictional personas and journey maps as a lens through which design and implementation considerations can be built.
4. Use of evaluative strategies: Students complete narrative reviews to assess readiness, as well as identify barriers and facilitators to implementing their proposed designs. A formal implementation blueprint is also developed and may include infographic posters, policy briefs, animations, booklets, and brochures suitable for use within their participating organizations.

### Phase 1: identify and recruit UREM AYA trainees for the introduction of the STAR institute

Key goals for phase 1 of STAR include: (1) the recruitment and program pool of trainees; (2) review and selection of team applications; and (3) retention of trainee teams.

#### Activity 1.1: recruitment and program Pool of trainees [crowdsourcing open calls (pre-summer training institute)]

Before the 6-week summer session starts, trainee team applicants respond to a crowdsourcing open call (see detailed description of open calls below), inviting trainees to participate in STAR. The purpose of the open call is to identify exceptional pairs of underrepresented minority trainees who are interested in implementation science, project-based design, and interdisciplinary HIV training and will join the STAR program. The call for trainees last for 1–2 months during which print and digital media are created and circulated to a continually updated list of programs throughout the institutions that serve minority students, including dedicated diversity enhancement personnel, academic departments, and career services programs, as well as all TRiO and Upward Bound Programs such as McNair programs, Latinos in Action, Black Student Union, Black Greek Organizations; and university chapters of the Society for the Advancement of Chicanos and American Indians in Science (SACNAS). The themes for the open calls align with national priorities for HIV programming focus on individuals and communities disproportionately affected by HIV. Submissions for the open call include short descriptions (<250 words) or images/videos in response to a specific prompt (for example, “How might we work with community organizations to promote HIV prevention services among youth aged 13–24 in your community?”) Our open calls are designed following standardized approaches to crowdsourcing as developed by the World Health Organization’s Special Programme for Research and Training in Tropical Diseases (19). A panel of 4 judges identifies three exceptional ideas from each institution in response to the open call, focusing on innovation, relevance to youth populations, and feasibility of implementation. Judges for the open call include young people, public health faculty and professionals, and community leaders. The top exceptional ideas from each participating institution are invited to move to the next phase: A 72-h designathon. At the end of the call, all contributions are screened for eligibility and judged using the following criteria:

1. Team composition: Each year, five pairs of trainees are selected, comprising graduate and/or undergraduate students from underrepresented minority backgrounds, with one person named the Team Lead in the application.
2. Submitted open call application packet: The teams’ application packet consists of a response to the crowdsourcing open call, a demographic application form, a resume/CV, and transcripts with GPA.
3. Open call ideas: Responses to the open calls are assessed for innovation, relevance to youth populations, and feasibility of implementation.
4. Research interests: All teams must submit a combined personal statement indicating their individual interests in pursuing a career in HIV-related research, implementation science, and commitment to completing the training program.
5. Virtual learning resources: Applicants complete a self-assessment to detail research and technology resources the teams have access to and can support the virtual learning experience.
6. Letter of recommendation: All applicants submit a letter of recommendation in support of team applications from faculty or community key stakeholders cognizant of the team’s research and career interests.

### The rationale for using crowdsourcing to recruit trainees

The rationale for using crowdsourcing to identify trainees is three-fold: First, accelerated efforts to diagnose, treat, and prevent HIV are urgently needed to achieve the U. S. goal of at least a 75% reduction in the number of new HIV infections by 2025 and 90% by 2030 (34). Generating wisdom from the crowd via crowdsourcing may accelerate the implementation of HIV testing strategies, such as the distribution of HIV self-testing kits to facilitate early diagnosis, thus achieving the first pillar of the Ending the HIV Epidemic: A Plan for America Initiative (35). Further, alternative methods along the HIV prevention continuum are needed to reach a large population of racial and ethnic minority youth in need for whom HIV prevention services are not available (36). Crowdsourcing is an emerging strategy that engages youth themselves to foster innovative and youth-informed ideas (11). As a form of youth-participatory action research, crowdsourcing invites the target users to develop and implement an intervention. To date, few programs give AYA the tools and resources necessary to lead the design and implementation of youth-friendly HIV interventions (8). This is due to a lack of training infrastructure, capacity to facilitate the development of research projects, and, most importantly, a lack of expertise to train the next generation of diverse investigators. To be optimally impactful, incorporating crowdsourcing with field implementation allows us to train and sustain a network of well-trained, diverse HIV researchers with complementary expertise to address the multifaceted and unique developmental needs of racial and ethnic minority youth in the US.

#### Activity 1.2: review and selection of team applications

All application responses to the open call are accepted via email or in person at participating institutions. After an initial screening for completeness and responsiveness, each application is reviewed by an independent STAR selection committee comprising four interdisciplinary judges (faculty engaged in HIV-related research, youth leaders, and key community stakeholders) with expertise or experience in HIV prevention programming to select candidates. The selection committee includes individuals from all participating institutions. Each submission is rated on a 1–10 scale, 10 being the highest score. After all scores are entered, compiled, and averaged, the selection committee meets to discuss and review top-ranking applicants. In general, 10 trainees (2-member teams) are selected to participate as a cohort.

#### Activity 1.3: retention of trainee teams

Since underrepresented racial and ethnic trainees are disproportionately vulnerable to being marginalized and structurally excluded from opportunities to remain in their biomedical training programs, we retain trainees using Simon Sinek’s “circle of safety” strategies (37). Specifically, we:

- Foster a sense of belonging with research via participatory learning communities and mentoring pods (see description in Aim 3), where concerns are addressed both on a team and one-on-one basis;
- Offer clear research requirements to encourage and propel interest in the field;
- Empower trainees to make decisions (i.e., presenting research findings at local or national conferences, organizing local exhibits of final interventions);
- Offer trust and empathy towards trainee life experiences as they occur.

### Phase 2: develop implementation science research and project-based design capacity for underrepresented trainees at participating U. S. Institutions

**Figure 2:**
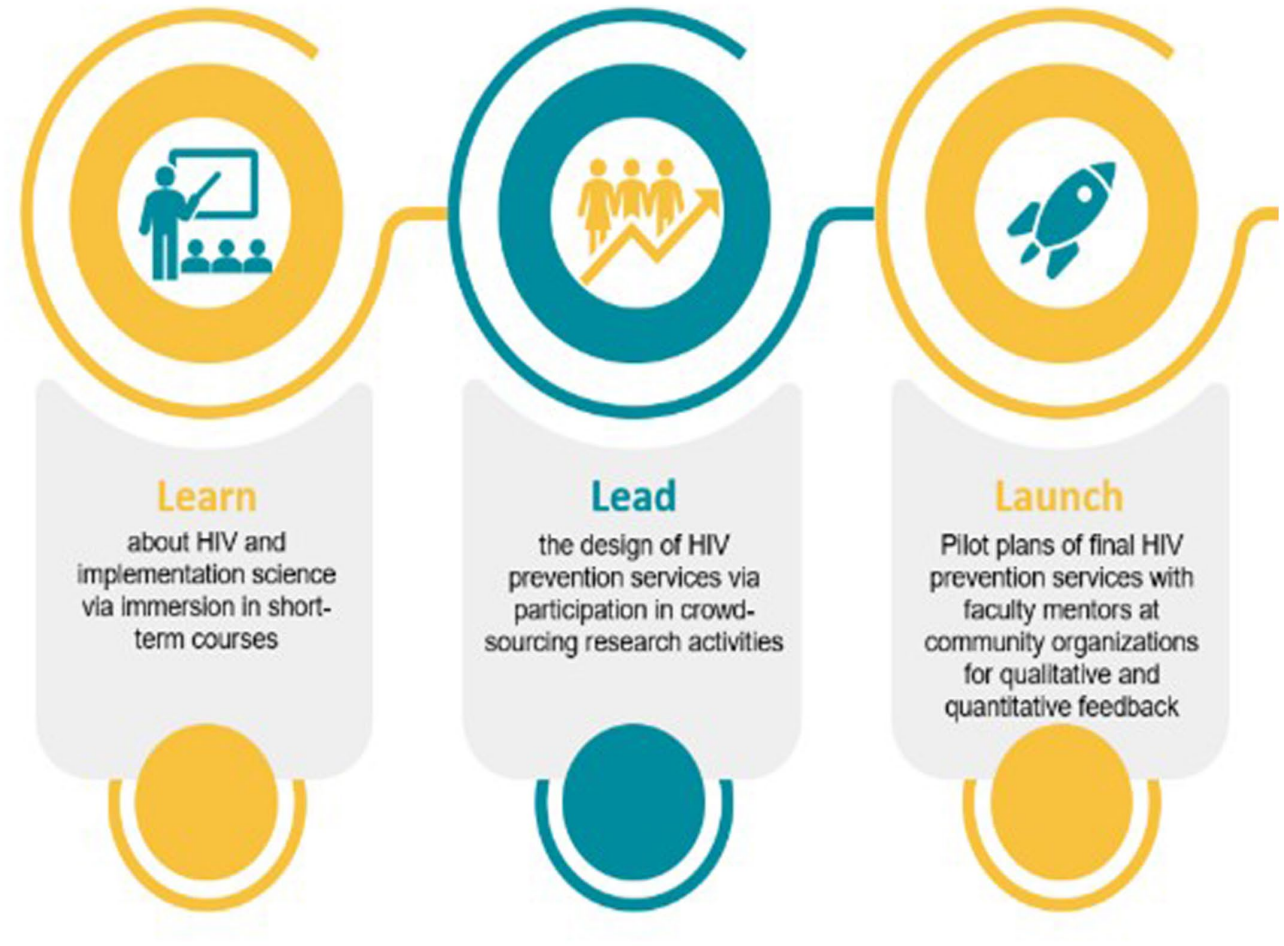
Key goals of the STAR program.

We train teams in implementation science research and project-based design, whereby students adapt and tailor their ideas to the local context. Key goals for this aim include: (1) Learning about HIV and implementation science as related to context assets and needs via immersion in short-term courses; (2) Leading the design of HIV prevention services via crowdsourcing and designathons tailored to local contexts; and (3) Launching the designed and finished products in collaboration with faculty mentors and partnerships with community organizations (Figure 2).

#### Activity 2.1: learning about HIV disparities and implementation science

We cultivate and deliver a short-term intensive implementation science research course. The curriculum for the intensive implementation science research follows the open-access Dissemination and Implementation Science Training Institute format at the NIH (38). The proposed STAR implementation science training uses the eight modules of the courses to guide Implementation Science concepts, frameworks, and principles in HIV prevention research. Moreover, the courses comprise a set of related topics delivered as didactic lectures in the morning. A dedicated faculty leads a variety of implementation science topics for undergraduate/graduate students as part of the short course module to include:

- Implementation Science Research and Resources
- Understanding Context and Population-Based HIV Prevention Landscape in the US
- Implementation theories, models, and frameworks
- Implementation strategies
- Working with different community partners
- Communication and Advocacy.
- Ethical research practices
- Role of Geospatial analysis with HIV programming.

The curriculum integrates CFIR and RE-AIM to guide both learning and project development. Trainees use CFIR to assess the context such as barriers and facilitators to implementation, while RE-AIM helps them evaluate the reach, adoption, and potential impact of their interventions. These frameworks are introduced during the short course and applied throughout the designathon, boot camp, and mentoring sessions as trainees refine their implementation blue-prints.

#### Activity 2.2: leading the design of HIV prevention services via crowdsourcing and Designathons tailored to local contexts

To refine project-based designs, we engage trainees in crowdsourcing from the onset via a set of activities, open calls for recruitment, and a designathon. Details are described below:

##### 72-h designathons (Pre-summer training institute)

A designathon is a sprint-like event that taps into diverse participant experiences and expertise to generate high-quality outputs transparently and systematically (39, 40). Designathons have been used to solve problems in education, technology, and public health (39, 40). It brings participants together to refine their proposed solutions developed during the open calls and leverages in-person workshops and teamwork to create holistic intervention packages. Compared with conventional, expert-driven approaches, designathons have greater community engagement, potential for innovation, and multisectoral collaboration. Meaningful youth involvement and engagement are emphasized as a key component of HIV campaigns targeting racial and ethnic minority youth in the US (36). Bringing together teams of trainees and community members from different sectors during the event enables a strong sense of community ownership. Furthermore, this approach can generate novel and efficient solutions that resonate with end users. The steps for our designathon are as follows:

- Kick-off: A lightning round panel where invited guest speakers share their experiences and answer questions about HIV prevention hurdles among racial and ethnic minority youth in the US.
- Brainstorming Activity: Teams participate in a structured brainstorming activity designed to uncover the problems from a youth perspective and identify possible solutions.
- Prototype: Over the next several hours, teams develop preliminary prototypes of their solutions and consult with rotating experts from the panel who answer teams’ questions and offer feedback.
- Pitch: Teams prepare pitch presentations lasting no more than 3 min, which are delivered to all designathon attendees, as well as a four-person panel of judges composed of youth, faculty, key leaders, and stakeholders in the field.

Judges evaluated the pitches on three categories: innovation, relevance to youth populations, and feasibility of implementation. Finalists from the designathon are invited to participate in the 6-week summer program.

#### Activity 2.3: launch the designed and finished products via innovation bootcamps

##### Innovation bootcamp (as part of a 6-week summer training institute)

The purpose of the innovation bootcamp is to build capacity for HIV prevention research targeting racial and ethnic minority youth over a several-week period. This immersive, project-based action-learning experience allows trainees to learn and work with key community partners to design contextually relevant projects that are applicable to the partners’ needs. The innovation bootcamp consists of three main components: (1) instructional hybrid (in-person and online via Zoom) seminars and workshops, (2) field trips, and (3) iterative concept/pitch development supported by mentors and facilitators. The instructional seminars and workshops lay the foundation for participants to engage in research and turn their ideas from the designathon into an HIV prevention service delivery strategy for racial and ethnic minority youth that could be implemented and tested for feasibility and acceptability. The concepts covered in the instructional seminars and workshops include simplifying research discussions with racial and ethnic minority youth using alternative strategies (journey mapping, personas), conducting local needs assessments, assessing readiness for change, identifying barriers and facilitators with change, and conducting field research. At the end of the academy, teams pitch their formal implementation blueprint, which may take the form of narrative reviews, infographic posters, policy briefs, animations, booklets, and brochures suitable for use within their participating organizations, and receive feedback from a panel of judges. The judges evaluate the blueprint based on innovation, relevance to youth populations, and feasibility of implementation. Through the boot camp, trainees interact with faculty and coaches who provide expertise, mentorship, and direction for proposed solutions during the bootcamp.

### Phase 3: initiate and sustain a virtual participatory learning community to facilitate collaboration between geographically dispersed trainees

Given that the project reinforces participatory and team-based learning, we convene a virtual learning workspace where teams meet to share experiences through presentations and creative writing or art expressions, participate in virtual webinars, attend online lectures, and collaborate with mentors and fellow trainee teams. Trainee teams discuss each other’s ideas and progress to better understand the process and use this structured opportunity to discuss their work. Key goals for this aim include: (1) Initiating participatory learning communities; (2) Facilitating reflection via creative writing, expressions, and presentation; and (3) Sustaining a network of STAR scholars.

#### Activity 3.1: initiate participatory learning communities

Modeled after the National Academy of Medicine Institute of Health Breakthrough Series (41), our advanced participatory learning communities provide a rich, collaborative environment to support trainees as they launch their pilots while fostering experiential learning at multiple levels as well as evaluation of findings from the exhibits. The PLCs include mentors and trainees to provide guidance as they launch pilots, evaluate preliminary acceptability and feasibility, and disseminate findings to a general audience. By initiating PLCs, this training program improves the chances of impacting research memory, creating continuity, and encouraging careers in biomedical research through experiential learning of project-related activities. Further, faculty and teams collaborate on other research opportunities to potentially stimulate additional future collaborations, including conducting pilots of their final interventions. PLCs also feature more time between trainees and mentors in multiple interactive workshops and learning sessions, with a focus on expanding on project ideas generated as part of STAR-wide activities.

#### Activity 3.2: facilitate reflection via creative writing, expressions, and presentations by individual trainees and cohorts

With the goal to enhance self-efficacy while encouraging careers in HIV research workforce, STAR facilitates reflection on experiences gained by each trainee through an annual journal for youth researchers by youth researchers. Reflection is ‘the purposeful, deliberate act of inquiry into one’s thoughts and actions through which a perceived problem is examined so that a thoughtful, reasoned response might be tested out” (42). Racial and ethnic trainees from underrepresented backgrounds may need to develop the skills to think and express themselves critically about their experiences, as well as their professional and personal competence in biomedical careers. Reflection, particularly through creative processes, can provide a vehicle for thinking about and acting on issues such as strengthening identity and self-efficacy as researchers. Reflection is utilized in the PLC to engage in critical dialogues with trainees about their experiences as STAR scholars and expectations for careers in the biomedical workforce. Trainees are asked to reflect on their experiences creatively using storytelling, poetry, journaling, or illustrative art. Findings are shared with peers via an annual STAR digital publication given to trainees at the end of their one-year program. Importantly, these activities have provided a sense of community, as defined by trainees, since they share a unique experience and have multiple expectations as future researchers and students in training.

#### Activity 3.3: sustain a network of STAR scholars

We facilitate and sustain a network of STAR scholars to enhance trainees’ professional development and nurture their career development and trajectory toward independent research in HIV and other related missions within NIAID. These networks are sustained through (1) quarterly virtual conversation café and (2) STAR social networking platforms (WhatsApp, LinkedIn, Twitter, Blogs) designed to help trainees, previous cohorts, and mentors maintain collaborations professionally. Conversation cafés are creative organizational or social design processes and a user-friendly tool for catalyzing conversations that enhance collective thought and lead to momentum for innovation (20). Every trainee is required to participate in an annual conversation café post-training to further share insights with past and new STAR cohorts regarding factors that predispose, facilitate, reinforce, and impede productive research careers. Similarly, we establish the STAR social networking platforms to sustain a network of STAR scholars. Users of the social networking platforms can utilize the platforms to chat with others in the network, send messages seeking advice, and share resumes, CVs, and publications. These low-cost strategies for sustaining engagement not only instill in trainees that they belong in the HIV research workforce but also broaden and translate their interests and abilities into meaningful career pursuits.

### Cross-cutting and transformative elements of STAR

At each of our four participating universities, one faculty member (program coordinator) is a primary mentor for STAR Trainee Teams from their respective institution. This mentorship is supported by the Multi-PIs (Drs. Iwelunmor and Tucker) as part of their roles as Project Directors. All mentors are accomplished researchers specializing in HIV infection, possessing diverse and complementary expertise. The trainee meets with the Mentoring Team at least every 2 months using Zoom or other means deemed appropriate by the group. Topics covered at these meetings include a review of the trainee’s progress in completing projects, assistance with publications, conference presentations, and career development plans. Racial and ethnic minority trainees continue to be underrepresented in leadership and influential positions in biomedical and behavioral careers as university deans and department chairs, NIH grant recipients, authors of peer-reviewed scientific journals, editors-in-chief, and editorial board members (43, 44). To address the underrepresentation of minority scholars in leadership roles, the program includes a leadership retreat led by Dr. Collins Airhihenbuwa and based on his research and leadership training entitled “Claim Your Space.” The objective of the training is based on the recognition that the path to true diversity in the scientific workforce requires a systems-level transformation that provides opportunities for underrepresented minorities to become leaders (45). Throughout the retreat, participants work in close-knit groups to foster reflection and draw inspiration from transformational leaders while synthesizing insights from session-wide activities. The retreat provides a comprehensive understanding of why leadership is crucial, showcases examples of transformational leadership across varying disciplines, and outlines the steps to claim your space, which includes ways to own your space, reaffirm your space, and envision your next space (45). Additionally, STAR trainees complete a grant-writing retreat during the 6-week summer research institute, combining lectures, hands-on activities, and discussions to demystify the NIH application process. A key focus is navigating the between-the-lines to improve the dialogue between grant writers and grant reviewers using practical, effective, and simple approaches developed in other fields. For example, the book *Purple Cow* by Seth Godin (46) illustrates how great specific aims should be well-crafted, effortless, and uniquely visible, almost like a purple cow among a group of brown cows in a grazing field. The retreat provides comprehensive training that supplies trainees with foundational tools to begin writing persuasive/effective grant proposals.

### Evaluation plan and success indicators

Program evaluation has been an integral part of the Institute from its commencement. The evaluation accomplishes three goals: (1) to provide data for decisions that affect the shaping and re-shaping (mid-course corrections) of the STAR program activities; (2) to determine the individual and aggregate success of program activities and aims; and (3) to generate the lessons learned and describe internal evidence-based programmatic decisions made throughout the evaluation processes.

### Baseline evaluation data

Baseline data from new trainees are obtained on program entry and entered into a REDCap database. Metrics include demographics such as gender, age, and education level; knowledge and skill level of research methods and ethics; CITI certification; experience with mentored research; number of prior publications (if any); and previous grants submitted and awarded (if any). We also track and collect all data required for entry into NIH training programs, such as contact, biographical, and training information, and trainee accomplishments (fellowships, awards, employment, product or policy developments, publications, funding received, presentations, posters at scientific conferences, and students taught or mentored).

### Trainee and team intake interview

The project team creates an online survey to ascertain trainee baseline competency in project-based design, Implementation Science research, interests, plans, and attitudes toward the training, careers, research, and educational environment.

### Individual development plans

With their primary faculty mentors, each trainee completes a formalized PLAN to explicitly outline their goals and areas of development during their time as a trainee. IDPs include statements about key people to collaborate with (i.e., circle of safety) alongside short-term learning and training goals as a trainee, long-term goals to monitor and/or adapt, strengths and weaknesses of the proposed plan, and specific action steps to sustain goals over time. IDPs also address ways to nurture and retain trainees within the STAR program, such as plans for continued participation with the journal clubs, reflections, conversation cafes, mentoring pods, and social networking opportunities. These plans monitor and adapt their training to meet individualized benchmarks for overall career development within the HIV research workforce.

### Team project presentations

As part of the STAR activities, teams present their final intervention plans to a panel of judges at the open call, designathon, and innovation bootcamps. These presentations occur in real time to a live audience and are broadcast to virtual participants for public feedback. We also provide written feedback from judges to each of the trainees using a standardized template.

### Annual evaluation of current and former trainees

STAR annually administers an online evaluation survey to all current and past trainees to assess the program’s progress. Metrics include demographics (gender, age, education); position, institution, and rank; number of publications (first author and total publications—this is also tracked by monitoring PubMed); the number of grant proposals submitted (as PI or co-I); the number of grants awarded; number of presentations at scientific conferences; number of new research partners and collaborations; and outcomes from their mentor relationships (satisfaction of relationship), sustained careers in HIV research, and activities with community partners. Current and former trainees are asked to provide an updated CV and NIH biosketch each year. Findings allow us to use the insights to iteratively improve STAR activities.

### Exit interviews

Upon program completion, all trainees participated in an “exit interview” to assess their progress and provide feedback for program improvement. During these interviews, trainees shared their post-integration training plans.

### Evaluation of short courses and additional training activities

After each training activity, trainees are asked to complete an evaluation of the event. Questionnaires assess the learning objectives of courses, quality of instruction, areas for improvement and unmet needs, and contribution to continued interest in HIV research, crowdsourcing, and implementation science. This feedback allows course content and structure to be modified if necessary.

### Overall assessment of STAR feasibility and acceptability via reflection activities

As part of the overall assessment of STAR feasibility and acceptability with diversifying the pipeline of the HIV research workforce, on an annual basis, we ask trainees to complete short written reflections of their overall assessment of STAR feasibility and acceptability, encouraging them to pursue careers within the HIV workforce. Specifically, participants are asked the following sample questions: (1) How did involvement in STAR impact you personally and professionally? What do you think about the training skills gained, the research conducted, or the mentoring received? What did you learn about yourself? What is your learning style, your leading style, and your project launch style? About yourself as a team member? What professional improvements have you made? Findings allowed us to evaluate STAR’s feasibility and acceptability while encouraging UREM trainees to pursue careers within the HIV research workforce.

### Long-term outcomes

In addition to our annual evaluation survey data on individual trainees, we have tracked our cumulative outcomes. These metrics include the total number of UREM students trained, trainee program retention and degree attainment (PhD, MSc, M. Phil.), and the number of trainees participating in Implementation Science courses and crowdsourcing activities. We track faculty appointments and NIH career development awards among postdoctoral fellows. Metrics on program research productivity include the total number of trainee scientific presentations at national and international scientific meetings, the total number of trainee peer-review publications, the total number of research grants submitted and received by trainees, position and academic rank for trainees after completion of training, and the total number of trainees who become mentors to new trainees. Qualitative data are collected and triangulated with survey data to conduct a comprehensive analysis of the program’s impact.

### Data analysis strategy

#### Quantitative analysis

Quantitative data collection through surveys, application forms, and program evaluations will be analyzed using descriptive and inferential statistics. Frequencies and proportions will summarize demographic variables and training outcomes, while means and standard deviations will describe continuous variables such as self-related competencies. Pre- and post-program assessments will be compared using paired statistical tests to evaluate changes in knowledge, self-efficacy, and research skills. Sample sizes will be calculated where relevant to assess the magnitude of change. Long-term indicators such as publications, grant submissions, and continued research engagement will be tracked annually and aggregated to assess program impact over time.

#### Qualitative analysis

Qualitative data from open-ended survey responses, exit interviews, and trainee reflections will be analyzed using an inductive thematic approach. Reviewers will independently read and code the data to identify recurring themes related to participant experiences, program strengths, and areas for improvement.

#### Mixed methods integration

Findings from quantitative and qualitative analyses will be integrated to provide a comprehensive evaluation of the STAR program. Quantitative outcomes such as improvements in knowledge or research productivity will be interpreted alongside narrative accounts that explain how these changes occurred. This triangulation of data will allow for a deeper understanding of the program’s feasibility, acceptability, and effectiveness in supporting underrepresented minority trainees in HIV research.

## Discussion

Racial and ethnic minority adolescents and young adults (AYA) aged 13–24 in the U. S. make up a significant proportion of people newly diagnosed with HIV (47, 48). However, they do not access essential HIV prevention services, including HIV testing, pre-exposure prophylaxis (PrEP), and sexually transmitted infection (STI) testing (1, 3, 49). This is partly explained by barriers that are individual (low perceived risk) (50), social (poor social support) (51), and structural (poor access to HIV testing and linkage to PrEP) (1, 52). Furthermore, the field of HIV prevention interventions lacks diversity, especially among underrepresented AYAs (53–56). Although underrepresented minority AYA represents a critical resource of talent that could be cultivated in efforts to expand the HIV research workforce, existing training programs are limited, and few institutions have innovative skills development, research experiences, and mentoring activities to support high-quality HIV training for this subgroup (53–56). Without an investment in the training and mentoring of underrepresented racial and ethnic minority (UREM) AYAs in HIV research, the national goals of expanding the pool of HIV research investigators from underrepresented backgrounds and ending the HIV epidemic by 2030 may remain elusive (35, 57, 58).

This STAR training institute presents a unique opportunity to use crowdsourcing, project-based design, and implementation science to diversify the research workforce, drive innovation, and increase health equity. The overall goal is to increase the pipeline of underrepresented minority students entering careers in HIV intervention research and education. By providing hands-on HIV research experience, skills development, and mentoring opportunities to undergraduate and graduate students, STAR aims to increase entry into and retention of trainees in HIV research, particularly those from backgrounds underrepresented in biomedical research. STAR also represents a novel model for enhancing diversity in the HIV research workforce. It combines crowdsourcing activities and several implementation science methods and strategies grounded in a youth participatory action research framework to advance the pipeline for careers in HIV research among racial and ethnic minority trainees (59, 60). We believe that tapping into the wisdom and lived realities of UREM AYA allows the identification of diverse and transformative solutions and decreases HIV disparities in the US (53–55). Bringing vibrant, diverse UREM trainees to the table is critical for addressing barriers to the uptake of essential HIV prevention services. It also ensures that the nation remains a global leader in scientific discovery and innovation via a pool of highly talented scientists from diverse backgrounds who help to further the strategic objectives of the NIH and NIAID (21). There are limitations worth mentioning with the proposed STAR program. First, there may be delays in the identification and enrollment of scholars. These delays may be related to competing priorities from AYA formal classwork or work-related obligations. We have extensive experience organizing crowdsourcing open calls for AYA using long periods of time (i.e., 2–3 months), as well as visits to classroom and community settings where students congregate to recruit participants. To address this, we expand promotion efforts in partnership with our community partners and institutional UREM champions. Regarding delayed enrollment, we collaborate with individual trainees to ensure we can provide sufficient support. Second, we anticipate that selected trainees may withdraw their participation over time. The STAR Trainees, including undergraduate students, graduate students, and postdoctoral fellows, are expected to be at an early career stage. We anticipate some instability in their career plans at this time. Examples include moving to another institution, taking additional classes or coursework over the summer, or withdrawing from school for individual health or family-related reasons. We work with the trainees to determine the best option. We replace the trainee if withdrawal occurs early in a training cycle. If withdrawal occurs later, we collaborate with the remaining trainees to ensure their training is not adversely affected.

## Conclusion

STAR addresses a critical gap in diversifying HIV research. By using project-based implementation science, which focuses on bottom-up strategies for youth engagement in HIV prevention research, we involve AYAs not as passive recipients or beneficiaries of deficit-minded interventions but rather as partners and leaders of strength-based HIV prevention interventions (9). Findings will build a needed evidence base for strategies that increase the adoption of essential HIV prevention services among at-risk UREM populations.

## Dissemination plans

Findings from the STAR program will be disseminated to key audiences through a variety of channels to maximize impact and accessibility. We will conduct dissemination meetings with academic and community stakeholders, using youth-friendly and culturally appropriate communication materials to increase accessibility for underrepresented minority adolescents and young adults (AYA). Results will be presented at national academic conferences focused on HIV, public health, implementation science, and published in Open Access, peer-reviewed journals that emphasize equity and behavioral science. To extend reach beyond academia, we will leverage social media platforms and programs and Participatory Learning Community (PLC) sessions to share program updates, outcomes, and youth-led innovations. Trainees will be supported in coauthoring presentations and publications, further promoting leadership and visibility. Datasets generated through the program will be made available upon completion of primary analysis and publication and may be requested by contacting the corresponding author.
